# The effect of uterocervical angle intervention on pain in retrovertion uterus: Evaluation of suprapubic compression: A retrospective observational study

**DOI:** 10.1097/MD.0000000000043692

**Published:** 2025-08-01

**Authors:** Melih Bestel, Elif Ucar, Salim Sezer, Suleyman Salman

**Affiliations:** a Department of Obstetrics and Gynecology, Istanbul Esenyurt University, Private Esencan Hospital, Istanbul, Turkey; b Department of Obstetrics and Gynecology, Health Sciences University, Gaziosmanpaşa Training and Research Hospital, Istanbul, Turkey.

**Keywords:** cervical canal, office hysteroscopy, pain management, suprapubic pressure, uterocervical angle, VAS score

## Abstract

This study aims to identify the factors affecting pain perception during office hysteroscopy and to evaluate the impact of suprapubic pressure on pain levels. In this retrospectively designed study, patients undergoing office-based “no-touch” hysteroscopy were evaluated. Participants were divided into 2 groups based on their pain scores using the Visual Analog Scale (VAS): VAS < 4 (low pain) and VAS ≥ 4 (high pain). Patient age, cervical canal passage time, presence of suprapubic pressure for uterocervical angle correction, and other demographic and clinical variables were analyzed. A significantly higher VAS score was observed in older patients and those with prolonged cervical canal passage time (*P* < .05). Contrary to expectations, pain scores were higher in the group where suprapubic pressure was applied. While a positive correlation was found between cervical passage time and VAS score, no significant relationship was detected between cervical canal length and VAS score. The most influential factor on pain perception during office hysteroscopy appears to be cervical canal passage time. Advanced age and increased difficulty in cervical access contribute to higher pain levels. Although suprapubic pressure may be applied to correct the uterocervical angle, it may increase discomfort due to its subjective nature. Therefore, pharmacological support may be considered for elderly patients, and future studies should aim to standardize pain-reducing interventions.

## 1. Introduction

Hysteroscopy is the gold standard method for evaluating the uterine cavity and obtaining endometrial tissue samples.^[1]^ Hysteroscopic surgery is widely used in the diagnosis and treatment of abnormal uterine bleeding, space-occupying lesions in the uterine cavity (such as polyps and myomas), infertility, uterine anomalies, and intrauterine adhesions.^[2]^ Office hysteroscopy, especially when performed using the “no-touch” technique, can often be conducted in an outpatient setting without the need for local or general anesthesia. These features not only allow avoidance of anesthesia-related complications and reduce costs, but also make the procedure quick, simple, and economical. As this technique does not require a vaginal speculum or cervical instruments, it helps minimize the pain typically associated with such tools. This increases patient tolerance, particularly among nulliparous individuals, sexually inactive women, and elderly patients with genital atrophy.^[3]^ Additionally, initiating the procedure with the vaginoscopic approach provides better visualization and enhances maneuverability within the uterine cavity.^[4]^ However, pain (often resulting from cervical manipulation or physical trauma) remains the most common reason for premature termination of the procedure.^[5]^ Furthermore, De Freitas et al found that office hysteroscopy is associated with less pain when performed by experienced surgeons (more than 500 cases).^[6]^ It is also important to consider that the perception of pain is subjective and varies according to each individual’s pain threshold.^[7]^ Given that pain is a key factor in discontinuing the procedure, it is essential to investigate its underlying causes and explore pre-procedural or intra-procedural interventions to minimize discomfort.

The angle between the cervical canal and the uterine axis is known as the uterocervical angle. Modifications to this angle may facilitate the hysteroscopic procedure and potentially reduce the risk of complications. Patients with retroverted uteri, a history of uterine surgery, atrophic tissue, or a narrow cervix are considered at higher risk for procedural difficulties and complications.

In this study, we aimed to investigate the factors influencing pain perception during office hysteroscopy. Additionally, we evaluated the potential advantages or disadvantages of applying simple suprapubic pressure, which may alter the uterocervical angle.

## 2. Materials and methods

This retrospective study evaluated 136 patients who underwent office hysteroscopy for various reasons at the Department of Obstetrics and Gynecology outpatient clinic of a tertiary care center between 2019 and 2020. The study protocol was approved by the institution’s ethics committee on October 6, 2021, under approval number 352.

Only patients with retroverted uteri detected by transvaginal ultrasound were included in the study. Patients who had previously undergone cervical surgery for benign or malignant reasons, those receiving treatment for vaginismus, those with vaginal infections, or those undergoing treatment for gynecological malignancies were excluded. Patients who underwent office hysteroscopy on odd days of the month received suprapubic compression, while those undergoing the procedure on even days of the month did not.

Before the hysteroscopy procedure, all patients underwent transvaginal ultrasound to confirm that the bladder was empty and that a retroverted uterus was present. The hysteroscopy was performed by the same surgeon using the no-touch technique. In this technique, no speculum or cervical instruments were used, and cervical dilation was not performed. During the procedure, a 3 mm rigid Olympus® instrument with a 30-degree optical lens was used. Saline infusion was administered, and intrauterine pressure was set at 75 mm Hg.

Suprapubic compression was applied immediately after vaginoscopy and before entering the cervical canal. The suprapubic pressure was gently applied over the uterocervical angle using the fist of the outer hand and continued throughout the procedure.

The most commonly used method for assessing pain was the Visual Analog Scale (VAS), applied to patients after the procedure.^[8]^ To evaluate the pain experienced by patients post-procedure, the VAS was used. The VAS is a 10 cm horizontal scale used on a card.^[8]^ Patients marked the level of pain they felt 15 minutes after the procedure on the card. Based on the marks, values between 0 and 10 were noted on the back of the card for the study.

### 2.1. Statistical analysis

Descriptive statistics for the data included mean, standard deviation, median, minimum, maximum, frequency, and percentage values. The distribution of variables was measured using the Kolmogorov–Smirnov test. The analysis of quantitative independent data was performed using Kruskal–Wallis, independent samples *t* test, and Mann–Whitney *U* test. The chi-square test was used for the analysis of qualitative independent data. Spearman correlation analysis was employed for correlation analysis. Statistical analyses were performed using SPSS 27.0 software.

## 3. Results

The study included 136 patients. The age of the patients ranged from 20 to 82 years. The characteristics of the population included in our study are shown in Table 1.

**Table 1:** Demographic characteristics of the patients.

Variable		Min–max	Median	Mean ± SD/n-%	
Age		20.0–82.0	430	42.3 ± 10.4	
Weight		43.0–110.0	740	73.8 ± 13.3	
Height		148.0–176.0	1.600	161.0 ± 5.5	
BMI		15.6–44.9	280	28.6 ± 5.7	
Gravida		0.0–13.0	300	3.08 ± 2.08	
Parity		0.0–3.0	200	1.82 ± 0.72	
Abortion		0.0–1.0	000	0.21 ± 0.41	
Curretage		0.0–3.0	000	0.20 ± 0.51	
	None			10	7.4%
Delivery	NSD			78	57.4%
	C/S			29	21.3%
	NSD + C/S			19	14.0%
Indication	DUB			35	25.7%
	Endometrial			94	69.1%
	Infertility			7	5.1%
Suprapubic	(−)			79	58.1%
Pressure	(+)			57	41.9%
Cervical canal transit time (s)		1.0–130.0	18.0	32.3 ± 33.4	
Cervical length (mm)		17.0–60.0	30.0	31.0 ± 6.2	
Period day		1.0–3.0	1.0	1.5 ± 0.8	
VAS		0.0–9.0	3.0	3.7 ± 2.2	

BMI = body mass index, C/s = C-section, DUB = dysfunctional uterin bleeding, max = maximum, min = minimum, mm = milimetres, NSD = normal spontane delivery, s = second, SD = standard deviation, VAS = Visual Analog Scale.

In this study, mode of delivery, indication for office hysteroscopy, suprapubic compression, cervical canal transit time, cervical length, menstrual period (1: follicular phase, 2: luteal phase, 3: postmenopausal period), and VAS score were used.

There was a significant (*P* < .05) positive correlation between VAS score and age and cervical canal transit time, but no significant (*P* > .05) correlation was observed between weight, height, body mass index, cervical length and gravidity, parity, abortion, and number of curettage (Table 2).

**Table 2:** Correlation of VAS score according to variables.

Variable		Age	Weight	Height	BMI	Cervical canal transit time (s)	Cervical length (mm)
VAS	r*	.189	.077	.113	.043	.471	.127
						.127	
	*P*	**.027**	.375	.190	.616	**.000**	.140
						.140	
Variable		Gravida	Parity	Abortion	Curretage	Period day	
VAS	r*	.142	.057	.105	.023	.063	
	*P*	.098	.507	.224	.793	.465	

Bold values indicate statistically significant correlations (*P* < .05). BMI = body mass index, mm = milimetres, s = second, VAS = Visual Analog Scale.

* Spearman correlation coefficient was used to analyze the relationship between 2 quantitative variables that do not have a normal distribution.

The mode of delivery and the indication for office hysteroscopy did not cause a significant (*P* > .05) change in the VAS score, but the VAS score was significantly (*P* < .05) higher in the group with suprapubic compression than in the group without suprapubic compression (Table 3).

**Table 3:** The effect of mode of delivery, indication for office hysteroscopy and presence of suprapubic compression on VAS.

		VAS			*P*
Variable		Min–max	Median	Mean ± SD	
Delivery	None	0.0–8.0	250	3.50 ± 2.635	
	NSD	0.0–9.0	300	3.31 ± 2.060	.113 K
	C/S	1.0–8.0	300	4.24 ± 2.182	
	NSD + C/S	1.0–7.0	400	4.37 ± 2.191	
Indication for office hysteroscopy	DUB	0.0–9.0	300	3.71 ± 2.444	
	Endometrial thickening	0.0–8.0	300	3.71 ± 2.103	.586 K
	Infertility	1.0–6.0	300	2.86 ± 1.773	
Suprapubic	(−)	0.0–8.0	300	3.27 ± 1.959	
Pressure	(+)	0.0–9.0	400	4.23 ± 2.345	**.021 **m
	K Kruskal–Wallis/m Mann–Whitney *U* test				

Bold values indicate statistically significant correlations (*P* < .05). C/S = C-section, DUB = dysfunctional uterin bleeding, max = maximum, min = minimum, NSD = normal spontane delivery, SD = standard deviation, VAS = Visual Analog Scale.

A positive, weak and statistically significant relationship was found between cervical canal transit time (seconds) and VAS in those without suprapubic compression (*r* = .443; *P* = .001). As the cervical canal transit time (seconds) increases in those without suprapubic compression, VAS will increase.

In patients with suprapubic compression, a positive, weak, and statistically significant relationship was found between cervical canal transit time (seconds) and VAS (*r* = .423; *P* = .001). In those without suprapubic compression, VAS will increase as the cervical canal transit time (seconds) increases.

In the total group, a positive, weak, and statistically significant relationship was found between cervical canal transit time (seconds) and VAS (*r* = .440; *P* < .001). As the cervical canal transit time (seconds) increases, VAS will increase (Table 4).

**Table 4:** Examination of the relationship between cervical canal transit time (s) and VAS according to suprapubic compression status and total group.

Variable*	Suprapubic pressure	VAS		
		No (n = 55)	Yes (n = 55)	Total (N = 110)
BMI	*r*	.132	.004	.059
	*P*	.336	.976	.543
Cervical canal passage time (s)	*r*	.443	.423	.440
	*P*	**.001**	**.001**	**<.001**

Bold values indicate statistically significant correlations (*P* < .05). BMI = body mass index, s = second, VAS = Visual Analog Scale.

* Spearman correlation coefficient was used to analyze the relationship between 2 quantitative variables that do not have a normal distribution.

In the group with VAS ≥ 4, the weight of the patients was observed to be higher than the group with VAS < 4. In addition, cervical canal transit time was statistically higher in the group with VAS ≥ 4. However, no significant difference was found between the 2 groups in terms of cervical length (Table 5).

**Table 5:** Comparison of general findings according to VAS categories.

VAS variable	<4		≥4		Statistical analysis* Possibility
	Mean ± SD	Median [IQR]	Mean ± SD	Median [IQR]	
Age (yr)	40.36 ± 11.49	40.0 [15.5]	44.19 ± 8.35	44.0 [11.5]	t = −1981 *P* = .051
Gravida	3.19 ± 2.55	2.0 [2.0]	3.17 ± 1.76	3.0 [2.0]	Z = −1077 *P* = .282
Parity	2.68 ± 2.09	2.0 [1.0]	2.56 ± 1.29	2.0 [1.0]	Z = −0.760 *P* = .447
Abortion	0.28 ± 0.99	0.0 [0.0]	0.36 ± 0.79	0.0 [0.5]	Z = −1135 *P* = .257
Curettage	0.19 ± 0.51	0.0 [0.0]	0.24 ± 0.58	0.0 [0.0]	Z = −0.628 *P* = .530
Weight	72.10 ± 14.35	73.0 [21.0]	77.26 ± 12.69	75.0 [16.0]	t = −1990 *P* = .049
Height	160.84 ± 5.18	160.0 [7.0]	161.24 ± 5.72	160.0 [7.0]	Z = −0.206 *P* = .837
BMI	28.01 ± 6.14	27.2 [8.4]	29.82 ± 5.31	29.4 [7.1]	t = −1652 *P* = .02
Cervical canal passage time (s)	18.68 ± 23.03	10.0 [14.0]	46.13 ± 40.27	35.0 [54.0]	Z = −4099 *P* < .001
CX (mm)	30.07 ± 6.71	30.0 [8.0]	31.26 ± 5.60	31.0 [6.5]	Z = −1899 *P* = .058
	n	%	n	%	
Delivery					
No delivery	5	8.8	4	7.5	*χ*^2^ = 3274
NSD	35	61.4	25	47.2	*P* = .351
C/S	11	19.3	13	24.5	
NSD + C/S	6	10.5	11	20.8	
Office indications					
DUB	11	19.3	15	28.3	*χ*^2^ = 5318
Endometrial	1	1.7	3	5.7	*P* = .378
thickening					
Polyp/myoma	39	68.4	30	56.6	
Infertility	4	7.0	2	3.7	
Isthmocele	1	1.8	–	–	
Uterine anomaly	1	1.8	3	5.7	
Suprapubic pressure					
Absent	33	57.9	22	41.5	*χ*^2^ = 2949
Present	24	42.1	31	58.5	*P* = .086
Cycle day					
Proliferative	39	68.4	33	62.3	*χ*^2^ = 0.462
Secretory	10	17.6	11	20.7	*P* = .794
Menopause	8	14.0	9	17.0	

BMI = body mass index, C/S = C-section, CX = cervix, mm = milimetres, NSD = normal spontane delivery, s = second, SD = standard deviation, VAS = Visual Analog Scale.

* Independent sample *t* test (*t*-table value) statistics were used to compare the measurement values of 2 independent groups for normally distributed data. Mann–Whitney *U* test (Z-table value) statistics were used to compare the measurement values of 2 independent groups in data that do not have a normal distribution. Pearson-*χ*^2^ cross tabulations were used to examine the relationship between 2 qualitative variables.

A statistically significant difference was found in terms of cervical canal transit time (seconds) according to VAS levels in those without suprapubic compression (*Z* = -2.806; *P* = .005). It was determined that the cervical canal transit time (seconds) of those with ≥ 4 VAS level was significantly higher than those with < 4 VAS level.

A statistically significant difference was found in terms of cervical canal transit time (seconds) according to VAS levels in patients with suprapubic compression (Z = -2943; *P* = .003). It was determined that the cervical canal transit time (seconds) of those with ≥ 4 VAS levels was significantly higher than those with < 4 VAS levels (Table 6).

**Table 6:** Comparison of cervical canal transit time (s) in terms of VAS levels according to suprapubic compression classes.

VAS levels Variable	<4		≥4		Statistical analysis* Mean ± SD
	Mean ± SD	Median [IQR]	Mean ± SD	Median [IQR]	
Suprapubic pressure [–]					
Cervical canal passage time (s)	20.15 ± 28.03	10.0 [15.0]	33.09 ± 26.81	27.5 [27.0]	Z = −2806 *P* = .005
Suprapubic pressure [+]					
Cervical canal passage time (s)	16.67 ± 13.84	13.5 [15.3]	55.38 ± 45.77	45.0 [100.0]	Z = −2943 *P* = .003

s = seconds, SD = standard deviation, VAS = Visual Analog Scale.

* Mann–Whitney *U* test (Z-table value) statistics were used in the comparison of 2 independent groups with measurement values in data that did not have a normal distribution.

## 4. Discussion

In this study, the pain experienced by patients undergoing office hysteroscopy and the factors affecting this pain were investigated. It was found that older age and a longer cervical canal transition time were significantly associated with higher pain levels. The suprapubic compression, which was applied in an attempt to modify the uterocervical angle and reduce the resulting pain, was observed to increase the pain felt by patients.

The “no-touch” technique in office hysteroscopy is a method that can be applied in outpatient settings and has the potential for implementing measures to increase patient comfort. During the “no-touch” hysteroscopy procedure, the pain typically felt is localized in the lower pelvic region and may be described as a burning, aching, or pressure-like sensation. This pain could result from cervical intervention, the passage through the cervical canal, uterine distension, or manipulation of the cavity during hysteroscopy. Interventions, such as cervical canal dilation, which increase the size of striated muscle, can lead to pain and discomfort. Additionally, pain may also arise from uterine contractions during the passage through the cervical canal.^[9]^ Therefore, minimizing cervical intervention, rapidly passing through the cervical canal, working with the lowest possible pressure to obtain a view, using the thinnest possible instruments, and moving the devices slowly and gently play significant roles in pain reduction. VAS scoring is commonly used in studies to assess the degree of pain felt by patients. In VAS scoring, a score of 2.5 to 3 is considered the upper limit of mild pain, while pain scores between 6.5 to 7 are defined as severe.^[10,11]^ In our study, we classified the patient group into 2 categories: VAS < 4 and VAS ≥ 4.

In older women or those in the menopausal period, conditions that may alter the uterocervical angle, change the position of the uterus, or cause cervical canal stenosis can complicate the hysteroscopy procedure. Additionally, Hameed et al reported that the likelihood of cervical laceration is more frequent in the older age group due to changes in the cervical collagen content.^[12]^ Güraslan et al observed moderate pain in postmenopausal and nulliparous women undergoing office hysteroscopy.^[13]^ In our study, we also found that the VAS score increased with older age. This may be attributed to an increase in cervical stenosis, narrowing of the cervical canal, and a decrease in uterine mobilization. Furthermore, the physical difficulties encountered during the procedure may increase the manipulation required by the performing physician, leading to increased pain. Therefore, in elderly patients, pain experienced during office hysteroscopy could potentially be alleviated with local anesthetics. Since pharmacological agents were not the subject of this study, further randomized controlled trials are needed to support our conclusion regarding the effectiveness of pharmacological agents in elderly patients.

Several studies have found a positive correlation between cervical canal passage time and VAS score. Soysal et al found that as the cervical canal transition time increased, the VAS score also increased. They also attributed the prolonged passage time to an increase in cervical canal length.^[14]^ In our study, it was observed that in the group with VAS scores ≥ 4, the cervical canal transition time was longer. Moreover, a positive correlation was found between cervical canal passage time and VAS score. However, no correlation was detected between cervical length and VAS score, and there was no significant difference in cervical length between the group with VAS ≥ 4 and the group with VAS < 4. No changes were observed with or without suprapubic compression applied to these variables. When all findings were evaluated together, it was considered that cervical canal manipulations were the primary cause of pain during the procedure. Additionally, due to the increased size of striated muscles from interventions, uterine contractions independent of cervical canal length are thought to contribute to increased pain levels.

Some studies have suggested that the uterine version/flexion may correlate with pain intensity in gynecological conditions like dysmenorrhea, dyspareunia, and chronic pelvic pain, thus highlighting the importance of uterine position.^[15]^ Cagnacci et al showed that dysmenorrhea was associated with anteverted uteri assessed by ultrasonography.^[16]^ Some studies have found no significant relationship between the uterine position and the pain level experienced by patients during diagnostic hysteroscopy.^[17]^ On the other hand, a study by Celik et al demonstrated that bladder distension increased the feasibility of hysteroscopy and reduced pain scores.^[18]^ It is thought that a full bladder may lead to important changes in pelvic anatomy. A full bladder applies mechanical anterior pressure on the uterus, which may temporarily change the position of the uterus to an anteverted position or create a more neutral axis, particularly in retroverted or narrow (<90°) uterocervical angles. This, in turn, may improve the angle between the cervix and the uterine cavity, potentially facilitating the procedure. Soysal et al aimed to straighten the uterocervical axis by applying external pressure to the suprapubic region in cases with a narrow uterocervical angle, which helped facilitate hysteroscope passage and reduce pain during the procedure. They observed that this approach provided distinct benefits, particularly in patients with a uterocervical angle below a certain threshold.^[14]^ Török et al sought to reduce pain during office hysteroscopy by applying suprapubic pressure. They suggested that this pressure could have a positive effect on the uterocervical angle in anteflexed uteri and reduce the pain experienced. However, they concluded that although this pressure did not significantly reduce pain, it made the procedure easier.^[5]^ In our study, we applied suprapubic pressure to correct the uterocervical angle in retroverted uteri to reduce pain during the procedure. However, upon reviewing the results, we observed higher VAS scores in the suprapubic pressure group. We believe this may be due to the patients’ inability to distinguish between the discomfort caused by suprapubic pressure and the discomfort caused by the hysteroscopy procedure itself. This led us to conclude that the pain related to the procedure might be perceived as less intense.

## 5. Limitations and prospects

Although suprapubic pressure was applied by the same individual in our study, it remains a subjective intervention. Additionally, the lack of measurement of the uterocervical angle before and after the application of suprapubic pressure represents a limitation of our study. Standardized intervention techniques, a more detailed examination of pain perception, and randomized studies based on uterocervical angle degrees may provide further insight in the future.

## 6. Conclusion

“No-touch” office hysteroscopy with minimal intervention in the cervical canal is a safe, fast and less painful procedure that does not require anesthesia. The main cause of pain in “no-touch” office hysteroscopy depends on the manipulation of the instrument during cervical canal passage and the speed of passage. Prolonged cervical canal passage time increases pain. It was observed that pain increased with increasing age and suprapubic compression to flatten the uterocervical axis had no effect on pain reduction in both cases. Randomized studies are needed to determine whether different interventions or pharmacological treatment is needed in the advanced age group or in patients with prolonged cervical canal passage.

## Author contributions

**Data curation:** Melih Bestel, Elif Ucar.

**Formal analysis:** Melih Bestel.

**Funding acquisition:** Elif Ucar.

**Investigation:** Melih Bestel.

**Methodology:** Melih Bestel.

**Project administration:** Elif Ucar.

**Resources:** Salim Sezer.

**Software:** Elif Ucar, Salim Sezer.

**Supervision:** Salim Sezer, Suleyman Salman.

**Validation:** Elif Ucar, Salim Sezer.

**Visualization:** Salim Sezer, Suleyman Salman.

**Writing – original draft:** Elif Ucar.

**Writing – review & editing:** Melih Bestel, Salim Sezer, Suleyman Salman.
